# Pubertal timing and incident uterine cancer in the Sister Study cohort

**DOI:** 10.1002/ijc.70310

**Published:** 2025-12-24

**Authors:** Ariayana N. Harrell, Katie M. O'Brien, Natalie D. Shaw, Dale P. Sandler, Mandy Goldberg

**Affiliations:** ^1^ Department of Sociology The University of North Carolina at Chapel Hill Chapel Hill North Carolina USA; ^2^ Epidemiology Branch National Institute of Environmental Health Sciences Research Triangle Park North Carolina USA; ^3^ Clinical Research Branch National Institute of Environmental Health Sciences Research Triangle Park North Carolina USA; ^4^ Department of Biostatistics and Epidemiology Rutgers School of Public Health Piscataway New Jersey USA

**Keywords:** endometrial cancer, menarche, puberty, thelarche, uterine cancer

## Abstract

Younger age at menarche is an established uterine cancer risk factor. Age at onset of breast development (thelarche), the earliest marker of pubertal “unopposed” estrogen exposure, may also be relevant to uterine cancer risk, particularly considering rapid declines in age at thelarche over time in parallel with increasing uterine cancer incidence rates. Using data from 34,152 participants with an intact uterus when they enrolled in the US Sister Study cohort (2003–2009; ages 35–74 years), we examined associations of self‐reported ages at thelarche and menarche with incident uterine cancer using multivariable‐adjusted Cox proportional hazards regression. We stratified by birth cohort, race, weight relative to peers in childhood, and body mass index (BMI) at enrollment to explore potential effect measure modification and tested for statistical heterogeneity across strata. During follow‐up (median = 13.3 years), 445 women reported an incident uterine cancer diagnosis. Ages at thelarche (hazard ratio [HR] per 1‐year older: 0.91, 95% confidence interval [CI]: 0.85–0.97) and menarche (HR per 1‐year older: 0.90, 95% CI: 0.84–0.96) were inversely associated with uterine cancer incidence. Associations were similar in non‐Hispanic Black and White women and did not vary by relative childhood weight or BMI in adulthood. Inverse associations of thelarche and menarche were limited to women born in 1950 or later (*p*‐het <.05). These findings suggest that younger ages at thelarche and menarche, markers of earlier and potentially prolonged exposure to estrogen in the absence of progesterone during puberty, may enhance susceptibility to uterine carcinogenesis.

## Graphical abstract

What's new?

Younger age at menarche is an established risk factor for uterine cancer. Age at onset of breast development (thelarche), the earliest marker of pubertal estrogen exposure unopposed by progesterone, may also be relevant to uterine cancer risk, but this association has not been explored. Using data from the US prospective Sister Study cohort, the authors found that earlier thelarche, as well as earlier menarche, were associated with increased rates of uterine cancer. These trends were particularly pronounced among women born after 1950. The findings suggest that secular declines in pubertal timing may contribute to the rising uterine cancer incidence rates.

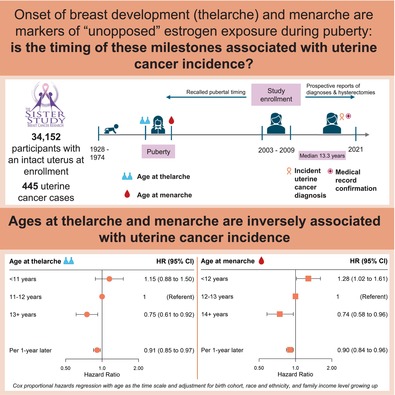

**Abbreviations**
BMI: body mass index
CI: confidence interval
HR: hazard ratio
SD: standard deviation

## 1 INTRODUCTION

Uterine cancer is the most common gynecologic cancer in the United States^1^ and second most commonly diagnosed gynecologic cancer globally.^2^ Endometrial cancer, which accounts for more than 90% of uterine cancers,^3^ is associated with hormone‐related factors across the life course.^4^, ^5^, ^6^ Uterine cancer incidence rates vary internationally—likely reflecting differences in fertility patterns, the prevalence of obesity, and other reproductive and lifestyle factors across geographic regions—with the highest rates observed in North America and Europe.^7^

The attainment of pubertal milestones marks hormonal changes in childhood and adolescence. Thelarche, the onset of breast development, is typically the first physical sign of pubertal estrogen exposure in girls.^8^ Thelarche occurs on average about 2–3 years before menarche, the first menstrual period.^9^ Menarche signals that sufficient estrogen has been produced to stimulate the growth of the endometrium. Notably, anovulatory cycles are common in the first few years after menarche and little progesterone is produced before ovulation^10^; thus, progesterone exposure remains minimal in the early gynecologic years.

The timing of these pubertal milestones and associated hormonal changes may be relevant to uterine cancer risk. Younger age at menarche has been associated with increased risk of endometrial cancer,^4^ potentially due to stimulation of endometrial cell proliferation resulting from longer exposure to “unopposed” estrogen (that is, estrogen without progesterone exposure), which increases susceptibility to carcinogenesis.^11^, ^12^ Thelarche represents an even earlier marker of pubertal unopposed estrogen exposure than menarche. To our knowledge, no studies have examined the timing of thelarche in relation to uterine cancer risk.

While age at menarche, on average, has declined little over the past 60 years, age at thelarche has been declining rapidly^13^; a global meta‐analysis estimated a mean decrease of almost 3 months per decade in age at thelarche from 1977 to 2013.^14^ In parallel, rates of uterine cancer incidence have been increasing over time,^7^, ^15^, ^16^ raising the possibility that the shift toward earlier thelarche has contributed to this increase.

Our objective was to examine associations of ages at thelarche and menarche with incident uterine cancer in the Sister Study, a US‐wide, prospective cohort study. We hypothesized that ages at thelarche and menarche would be inversely associated with uterine cancer incidence. In secondary analyses, we examined joint effects of timing of thelarche and menarche and explored race, birth cohort, and body size—factors associated with pubertal timing^13^, ^17^, ^18^, ^19^—as potential modifiers of the relationship between ages at pubertal milestones and incident uterine cancer.

## 2 MATERIALS AND METHODS

### 2.1 Study population

Women were eligible to join the Sister Study if they fit these criteria at enrollment: did not have a personal history of breast cancer, had at least one sister with a history of breast cancer, lived in a US state or Puerto Rico, and were between the ages of 35–74 years (for additional details, see Ref. [20]). From 2003 to 2009, 50,884 participants enrolled in the study. Women completed an extensive computer‐assisted baseline telephone interview. They are followed prospectively with annual health updates and more comprehensive follow‐up questionnaires every 2–3 years. Response rates have been 80% or higher throughout the follow‐up period. This analysis used Sister Study Data Release 11.1, including follow‐up through September 30, 2021.

### 2.2 Analytic sample

Out of 50,884 Sister Study participants, we excluded women who withdrew their data (*n* = 5), were diagnosed with uterine cancer prior to study enrollment or at an unknown time relative to enrollment (*n* = 415), had an uncertain uterine cancer diagnosis (*n* = 16), reported a hysterectomy prior to enrollment (*n* = 15,594), had no follow‐up data (*n* = 200), had missing (*n* = 403) or implausibly late puberty information (*n* = 31), which we defined as age at thelarche ≥21 years or age at menarche ≥22 years, or who were missing data on key covariates (*n* = 68) (Figure S1). The analytic sample therefore consisted of 34,152 women.

### 2.3 Pubertal timing assessment

At the baseline interview, participants reported the age in years when they first noticed that their breasts started to develop (thelarche) and the age in years and months when they experienced their first menstrual period (menarche). If age was unknown, women were asked to report their grade in school or, for menarche, their timing relative to peers; we converted these responses to ages as described previously.^21^ We examined ages at thelarche and menarche as continuous variables and categorized the timing of each as early, average, and late based on ages at these milestones from prior studies spanning similar birth cohorts (reviewed in Ref. [18]) and the distribution in our data (thelarche: <11, 11–12, and ≥13 years; menarche: <12, 12–13, and ≥14 years).

### 2.4 Outcome assessment

Incident uterine cancer cases were defined based on a self‐, next‐of‐kin, or death record report of an endometrial cancer, uterine sarcoma, or other type of uterine cancer diagnosed after enrollment. Participants who self‐reported a uterine cancer were asked to provide authorization to obtain pathology reports to confirm the diagnosis and obtain histology information. A total of 349 cases were confirmed based on data from pathology reports (*n* = 326) or death certificates or National Death Index Plus (*n* = 23). Given a positive predictive value of 80% among self‐reported cases who provided pathology reports, we considered all remaining self‐ (*n* = 92) and next‐of‐kin (*n* = 4) reported uterine cancers to be cases for our main analysis. Among medically confirmed endometrial cancers, we used histology codes, when available, to classify tumors into the histologic subtypes of endometrioid and nonendometrioid tumors.^16^

### 2.5 Covariate assessment

We categorized participant's birth year into approximately 10‐year intervals (1928–1939, 1940–1949, 1950–1959, and 1960–1974). At the baseline interview, participants self‐identified their race using provided categories (American Indian or Alaska native, Asian, Black or African American, Native Hawaiian or other Pacific Islander, and/or White) and reported if they considered their ethnicity to be Hispanic or Latina. We categorized race and ethnicity for this analysis as non‐Hispanic White, non‐Hispanic Black, Hispanic/Latina, and a group consisting of all others due to small numbers. Participants self‐reported their family income level growing up as poor, low income, middle income, or well‐off. Participants also reported their weight relative to peers at age 10 years (lighter, same weight, or heavier). We used measurements of height and weight taken by an examiner at the baseline home visit to calculate body mass index (BMI) at enrollment. We did not include well‐known uterine cancer risk factors that occur after puberty (e.g., parity, age at first birth, and exogenous hormone use) as covariates because these factors cannot be confounders but rather are potential mediators of our associations of interest.

### 2.6 Statistical analyses

We examined the distributions of participant characteristics in the analytic sample and among incident uterine cancer cases. We used Cox proportional hazards regression with age as the time scale to estimate hazard ratios (HR) and 95% confidence intervals (CI) for the associations of ages at thelarche and menarche with incident uterine cancer. Participants accrued person‐time from age at study entry until age at uterine cancer diagnosis, with censoring at age at hysterectomy, last study follow‐up, loss to follow‐up, or death. In multivariable‐adjusted models, we included terms for birth cohort, race and ethnicity, and family income level growing up. We examined ages at thelarche and menarche continuously and in categories. In primary models, we examined age at each milestone separately due to their positive correlation. We also present a model mutually adjusted for ages at thelarche and menarche. We further considered the timing of thelarche and menarche jointly using a 9‐category variable (combinations of early, average, or late ranging from both early to both late).

We did not include weight relative to peers at age 10 years as a covariate in our primary models because it could potentially be a confounder or a mediator of associations of ages at thelarche and menarche with uterine cancer incidence, depending on the timing of weight assessment relative to the timing of puberty. However, we present estimates from secondary analyses additionally adjusting for (1) relative childhood weight (as a categorical variable) and (2) BMI at enrollment (as a continuous variable). BMI in adulthood is a well‐recognized uterine cancer risk factor^22^ that is correlated with childhood weight,^23^ the true confounder of interest, and is a potential mediator.

We conducted sensitivity analyses restricted to medically confirmed uterine cancer cases and medically confirmed endometrial cancer cases to evaluate the robustness of our findings across different outcome definitions. Among medically confirmed endometrial cancer cases, we examined endometrioid and nonendometrioid tumors separately, censoring uterine cancer cases that did not meet the specific subtype definition at the age of their diagnosis.

We examined effect measure modification by race (non‐Hispanic White, non‐Hispanic Black), birth cohort (before 1950, 1950, or later), weight relative to peers at age 10 years (lighter, same, or heavier), and BMI at enrollment (<25, ≥25 kg/m^2^) through stratification and tested for statistical heterogeneity using a Wald test of all exposure‐by‐modifier interaction terms. We also conducted analyses stratified by time‐varying menopausal status and age (≤60, >60 years).

We conducted additional analyses to consider the sensitivity of the estimated association of age at thelarche with uterine cancer incidence to potential errors in the recall of age at thelarche. Biologically, we expect thelarche to occur before menarche. For the first set of sensitivity analyses, we therefore assumed that anyone recalling thelarche <1 year before menarche actually experienced thelarche earlier and imputed age at thelarche for those participants by either (1) re‐assigning their age to be first 1 and then 2 years earlier than originally reported or (2) setting age at thelarche to missing and using a multiple imputation (MI) model. For the latter, we generated 20 datasets with age at thelarche imputed using chained equations with the predictive mean matching method, including uterine cancer status, crude cumulative hazard estimates, age at menarche, birth cohort, race and ethnicity, family income level growing up, relative weight at age 10 years, BMI at enrollment, and early‐life exposures that we previously found to be associated with age at thelarche^24^ in the imputation model (see Table S1 for more details). In the second set of sensitivity analyses, we restricted the sample to informative subgroups. First, we examined participants who reported that thelarche occurred at least 1 year before menarche (*n* = 15,846). Next, to consider the influence of extremely early and late recalled ages, we restricted the analysis to participants who reported thelarche at ages 8–14 years (*n* = 31,632). Finally, we examined the thelarche‐uterine cancer association among participants who reported menarche at 13 years of age (the median age at menarche in the cohort, *n* = 10,059). The purpose here was to isolate the independent contribution of age at thelarche from that of age at menarche.

We do not present estimates for groups with fewer than five exposed cases. We used cluster robust sandwich covariance estimates^25^ to account for within‐family correlations, as multiple sisters from the same family could be included. We conducted analyses using SAS version 9.4 and created figures in R version 4.2.3.

## 3 RESULTS

During a median follow‐up time of 13.3 years, 445 eligible participants reported an incident uterine cancer diagnosis. The mean age at baseline was 54.1 years (standard deviation [SD] = 8.9) in the analytic sample and 57.4 years (SD = 8.0) among uterine cancer cases (Table 1). The mean ages at thelarche and menarche were 12.2 years (SD = 1.6) and 12.7 years (SD = 1.5), respectively, in the analytic sample. Among uterine cancer cases, mean ages at thelarche and menarche were 12.0 years (SD = 1.6) and 12.4 years (SD = 1.4). The largest proportion of participants identified as Non‐Hispanic White (85%), were born between 1950 and 1959 (39%), grew up in a middle‐income household (62%), and reported that they were the same weight as their peers at age 10 years (47%).

**TABLE 1:** Characteristics of eligible Sister Study participants overall (*n* = 34,152) and among uterine cancer cases (*n* = 445).

	Full analytic sample		Uterine cancer cases	
	(*N* = 34,152)		(*N* = 445)	
	Mean	SD	Mean	SD
Age at baseline (years)	54.1	8.9	57.4	8.0
Age at thelarche (years)	12.2	1.6	12.0	1.6
Age at menarche (years)	12.7	1.5	12.4	1.4

	*n*	%	*n*	%
Birth cohort				
1928–1939	3033	9	63	14
1940–1949	9889	29	167	38
1950–1959	13,417	39	175	39
1960–1974	7813	23	40	9
Race/ethnicity				
Non‐Hispanic White	29,080	85	381	86
Non‐Hispanic Black	2577	8	39	9
Hispanic	1640	5	19	4
All others^a^	855	3	6	1
Family income level growing up				
Well off	2356	7	31	7
Middle income	21,281	62	286	64
Low income	8284	24	87	20
Poor	2231	7	41	9
Weight relative to peers at age 10				
Lighter	11,851	35	136	31
Same	15,908	47	208	47
Heavier	6308	19	98	22
Missing	85		3	
Body mass index at enrollment				
<25 kg/m^2^	14,551	43	106	24
≥25 kg/m^2^	19,590	57	339	76
Missing	11		0	

*Note*: Column percentages are displayed. Missing data are excluded from percentages. Percentages may not add up to 100 due to rounding.

Abbreviation: SD, standard deviation.

^a^ Participants who identified as non‐Hispanic and Asian, Native Hawaiian or other Pacific Islander, and/or American Indian or Alaska Native. This category is not further disaggregated to protect participant confidentiality due to small case counts.

In multivariable‐adjusted models, the uterine cancer HR for early thelarche (<11 years) was 1.15 (95% CI: 0.88–1.50) compared to experiencing thelarche at ages 11–12 years, while the HR for late thelarche (≥13 years) was 0.75 (95% CI: 0.61–0.92) (Table 2). The HR was 1.28 (95% CI: 1.02–1.61) for early menarche (<12 years) and 0.74 (95% CI: 0.58–0.96) for late menarche (≥14 years) compared to menarche at ages 12–13 years. When modeled continuously, ages at thelarche and menarche were both inversely associated with uterine cancer incidence (HR 0.91, 95% CI 0.85–0.97 per 1‐year older thelarche and 0.90, 95% CI 0.84–0.96 per 1‐year older menarche).

**TABLE 2:** Hazard ratios (HRs) and 95% confidence intervals (CIs) for the associations of timing of pubertal milestones with incident uterine cancer in the Sister Study cohort (*n* = 34,152 participants).

Pubertal milestone	Person‐years	*n* Cases	Age‐adjusted^a^		Multivariable‐adjusted^b^		Mutually adjusted^c^	
			HR	95% CI	HR	95% CI	HR	95% CI
Age at thelarche								
<11 years	53,210	68	1.16	0.89, 1.52	1.15	0.88, 1.50	1.04	0.77, 1.41
11–12 years	203,068	235	1	Referent	1	Referent	1	Referent
≥13 years	166,161	142	0.75	0.61, 0.92	0.75	0.61, 0.92	0.83	0.67, 1.04
Continuous (per 1‐year older)	422,439	445	0.91	0.85, 0.97	0.91	0.85, 0.97	0.95	0.88, 1.03
Age at menarche								
<12 years	78,374	109	1.29	1.03, 1.61	1.28	1.02, 1.61	1.20	0.93, 1.56
12–13 years	242,747	258	1	Referent	1	Referent	1	Referent
≥14 years	101,318	78	0.74	0.58, 0.96	0.74	0.58, 0.96	0.81	0.62, 1.05
Continuous (per 1‐year older)	422,439	445	0.89	0.84, 0.95	0.90	0.84, 0.96	0.93	0.85, 1.00

^a^ Adjusted for age as the time scale.

^b^ Additionally adjusted for birth cohort, race and ethnicity, and family income level growing up.

^c^ Multivariable‐adjusted model including ages at thelarche and menarche (both as categorical variables or both as continuous variables).

**FIGURE 1:**
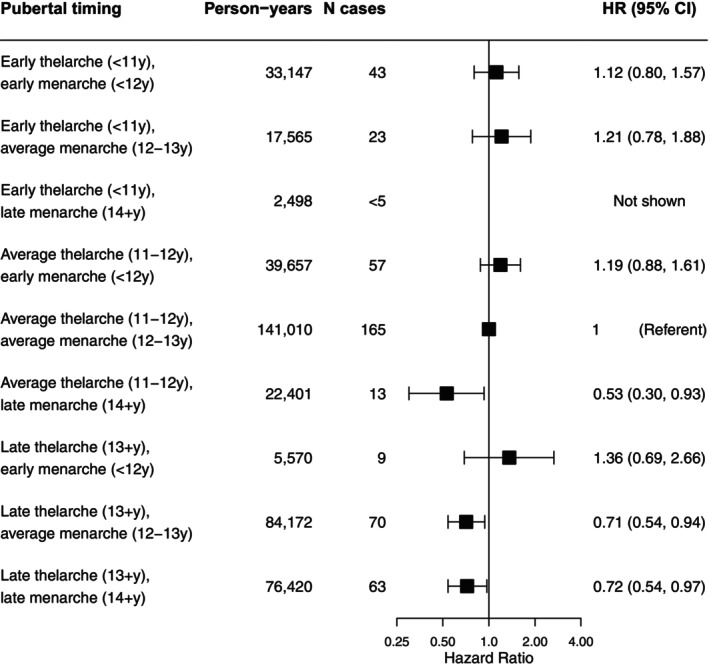
Hazard ratios and 95% confidence intervals (CIs) for the associations of joint timing of thelarche and menarche with incident uterine cancer in the Sister Study cohort (*n* = 34,152 participants). Estimates are adjusted for age as the time scale, birth cohort, race and ethnicity, and family income level growing up. Estimates are not presented for categories with less than five exposed cases.

Ages at thelarche and menarche were moderately correlated (Spearman's rho: 0.6), and associations were somewhat attenuated in mutually adjusted models (Table 2). When we considered the timing of thelarche and menarche jointly relative to experiencing both at average ages, HRs suggested that an older age at either milestone was associated with decreased incidence (Figure 1). HRs were generally positive in direction for groups characterized by early thelarche or menarche, but CIs were wide. There were few participants who reported early age at one milestone and late age at the other. The HR for the late thelarche and early menarche group was positive but imprecise, aligning with the trend for the timing of menarche.

HRs were similar when adjusted for relative weight at age 10 but attenuated when additionally adjusted for BMI at enrollment (Table 3). Patterns of associations with ages at thelarche and menarche were similar in analyses restricted to medically confirmed cases of uterine cancer, endometrial cancer, endometrioid endometrial cancers, and nonendometrioid endometrial cancers (Table 4).

**TABLE 3:** Hazard ratios (HRs) and 95% confidence intervals (CIs) for the associations of timing of pubertal milestones with incident uterine cancer in the Sister Study cohort additionally adjusted for childhood and adult body size (*n* = 34,056 participants^a^).

Pubertal milestone	Person‐years	*n* Cases	Multivariable‐adjusted model + relative weight at age 10 years^b^		Multivariable‐adjusted model + relative weight at age 10 years + BMI at enrollment^c^	
			HR	95% CI	HR	95% CI
Age at thelarche						
<11 years	53,119	67	1.09	0.83, 1.44	1.00	0.76, 1.33
11–12 years	202,460	234	1	Referent	1	Referent
≥13 years	165,757	141	0.77	0.62, 0.95	0.81	0.66, 1.00
Continuous (per 1‐year older)	421,336	442	0.92	0.87, 0.99	0.95	0.89, 1.02
Age at menarche						
<12 years	78,184	108	1.24	0.99, 1.56	1.13	0.89, 1.42
12–13 years	242,154	256	1	Referent	1	Referent
≥14 years	100,998	78	0.77	0.60, 1.00	0.81	0.63, 1.05
Continuous (per 1‐year older)	421,336	442	0.91	0.85, 0.97	0.95	0.89, 1.01

Abbreviation: BMI, body mass index.

^a^ These models include 34,056 participants (*n* = 96 participants [three incident uterine cancer cases and 93 non‐cases] were excluded due to missing data on relative childhood weight or BMI at enrollment).

^b^ Adjusted for age as the time scale and birth cohort, race and ethnicity, family income level growing up, and weight relative to peers at age 10 years as covariates.

^c^ Includes all in (b) plus BMI at enrollment as a continuous covariate.

**TABLE 4:** Hazard ratios (HRs) and 95% confidence intervals (CIs) for the associations of timing of pubertal milestones with medically confirmed incident uterine cancer in the Sister Study cohort (*n* = 34,152 participants).

	Age at thelarche											
	<11 years			11–12 years			≥13 years			Continuous (per 1‐year older)		
	*n* Cases	HR^a^	95% CI	*n* Cases	HR^a^	95% CI	*n* Cases	HR^a^	95% CI	*n* Cases	HR^a^	95% CI
All uterine cancer	68	1.15	0.88, 1.50	235	1	Referent	142	0.75	0.61, 0.92	445	0.91	0.85, 0.97
Medically confirmed uterine cancer	61	1.35	1.01, 1.80	181	1	Referent	107	0.74	0.58, 0.94	349	0.87	0.81, 0.93
Medically confirmed endometrial cancer	54	1.30	0.96, 1.77	167	1	Referent	100	0.75	0.58, 0.96	321	0.88	0.81, 0.94
Endometrioid endometrial cancer	44	1.31	0.93, 1.84	135	1	Referent	81	0.75	0.57, 0.99	260	0.88	0.81, 0.95
Nonendometrioid endometrial cancer	8	1.44	0.65, 3.19	23	1	Referent	14	0.77	0.40, 1.49	45	0.84	0.70, 1.00

	Age at menarche											
	<12 years			12–13 years			≥14 years			Continuous (per 1‐year older)		
	*n* Cases	HR^a^	95% CI	*n* Cases	HR^a^	95% CI	*n* Cases	HR^a^	95% CI	*n* Cases	HR^a^	95% CI
All uterine cancer	109	1.28	1.02, 1.61	258	1	Referent	78	0.74	0.58, 0.96	445	0.90	0.84, 0.96
Medically confirmed uterine cancer	93	1.45	1.13, 1.85	196	1	Referent	60	0.76	0.57, 1.01	349	0.87	0.81, 0.94
Medically confirmed endometrial cancer	83	1.37	1.06, 1.78	185	1	Referent	53	0.71	0.52, 0.96	321	0.88	0.81, 0.94
Endometrioid endometrial cancer	65	1.29	0.97, 1.73	155	1	Referent	40	0.64	0.45, 0.90	260	0.87	0.80, 0.94
Nonendometrioid endometrial cancer	16	2.29	1.19, 4.40	21	1	Referent	8	0.97	0.43, 2.17	45	0.85	0.66, 1.10

*Note*: Non‐medically confirmed uterine cancers and alternate types, when applicable, were censored at age at diagnosis.

^a^ Adjusted for age as the time scale, birth cohort, race and ethnicity, and family income level growing up.

We observed heterogeneity in uterine cancer HRs for both thelarche and menarche when we stratified by birth cohort (Table 5). In continuous exposure models, we observed inverse associations of ages at thelarche and menarche with incident uterine cancer only among participants born in 1950 or later. In models stratified by time‐varying menopausal status and age, HRs in continuous exposure models were stronger for pre‐menopausal women and women ≤60 years for both thelarche and menarche. Associations of ages at thelarche and menarche with uterine cancer were similar among non‐Hispanic White and non‐Hispanic Black women and did not differ by weight relative to peers at age 10 or BMI at enrollment.

**TABLE 5:** Hazard ratios (HRs) and 95% confidence intervals (CIs) for the associations of timing of pubertal milestones with incident uterine cancer within strata of potential modifiers of interest in the Sister Study cohort (*n* = 34,152 participants).

	Age at thelarche													
	<11 years			11–12 years			≥13 years				Continuous (per 1‐year older)			
	*n* Cases	HR^a^	95% CI	*n* Cases	HR^a^	95% CI	*n* Cases	HR^a^	95% CI	*p*‐het^b^	*n* Cases	HR^a^	95% CI	*p*‐het^b^
Birth cohort										.09				.02
Before 1950	27	0.97	0.64, 1.48	120	1	Referent	83	0.88	0.66, 1.16		230	0.99	0.91, 1.07	
1950 or later	41	1.28	0.89, 1.83	115	1	Referent	59	0.62	0.45, 0.85		215	0.84	0.77, 0.93	
Menopausal status										.56				.17
Pre‐menopausal	7	1.06	0.46, 2.47	24	1	Referent	11	0.52	0.25, 1.05		42	0.80	0.67, 0.96	
Post‐menopausal	61	1.16	0.87, 1.54	211	1	Referent	131	0.78	0.62, 0.96		403	0.92	0.86, 0.99	
Age										.69				.24
≤60 years	25	1.32	0.83, 2.10	68	1	Referent	41	0.72	0.49, 1.06		134	0.86	0.76, 0.97	
>60 years	43	1.07	0.76, 1.49	167	1	Referent	101	0.76	0.59, 0.97		311	0.93	0.87, 1.01	
Race and ethnicity										.78				.69
Non‐Hispanic Black	9	1.48	0.65, 3.35	16	1	Referent	14	0.95	0.46, 1.96		39	0.94	0.75, 1.18	
Non‐Hispanic White	56	1.17	0.87, 1.57	204	1	Referent	121	0.75	0.60, 0.93		381	0.90	0.84, 0.97	
Weight relative to peers at age 10 years^c^										.99				.99
Lighter	11	1.12	0.59, 2.14	62	1	Referent	63	0.84	0.59, 1.19		136	0.92	0.83, 1.03	
Same	28	1.07	0.71, 1.62	119	1	Referent	61	0.74	0.54, 1.00		208	0.93	0.84, 1.03	
Heavier	28	1.09	0.69, 1.71	53	1	Referent	17	0.73	0.42, 1.25		98	0.92	0.79, 1.07	
BMI at enrollment^d^										.79				.45
<25 kg/m^2^	13	1.30	0.70, 2.40	52	1	Referent	41	0.85	0.57, 1.28		106	0.89	0.78, 1.02	
≥25 kg/m^2^	55	1.03	0.77, 1.40	183	1	Referent	101	0.77	0.61, 0.98		339	0.95	0.88, 1.02	

	Age at menarche													
	<12 years			12–13 years			≥14 years				Continuous (per 1‐year older)			
	*n* Cases	HR^a^	95% CI	*n* Cases	HR^a^	95% CI	*n* Cases	HR^a^	95% CI	*p*‐het^b^	*n* Cases	HR^a^	95% CI	*p*‐het^b^
Birth cohort										.003				<.001
Before 1950	45	0.95	0.68, 1.33	136	1	Referent	49	0.91	0.65, 1.26		230	1.00	0.92, 1.09	
1950 or later	64	1.69	1.25, 2.28	122	1	Referent	29	0.57	0.38, 0.86		215	0.80	0.73, 0.87	
Menopausal status										.18				.03
Pre‐menopausal	12	1.51	0.75, 3.03	26	1	Referent	<5	NS^e^			42	0.73	0.61, 0.88	
Post‐menopausal	97	1.26	0.99, 1.60	232	1	Referent	74	0.80	0.61, 1.04		403	0.91	0.86, 0.98	
Age										.06				.005
≤60 years	35	1.37	0.92, 2.03	83	1	Referent	16	0.45	0.26, 0.77		134	0.78	0.70, 0.87	
>60 years	74	1.25	0.95, 1.65	175	1	Referent	62	0.89	0.67, 1.20		311	0.95	0.88, 1.02	
Race and ethnicity										.85				.43
Non‐Hispanic Black	12	1.51	0.73, 3.09	20	1	Referent	7	0.91	0.39, 2.15		39	0.96	0.78, 1.17	
Non‐Hispanic White	89	1.26	0.98, 1.61	225	1	Referent	67	0.74	0.56, 0.97		381	0.88	0.82, 0.95	
Weight relative to peers at age 10 years^c^										.98				.92
Lighter	21	1.22	0.75, 1.99	77	1	Referent	38	0.83	0.56, 1.23		136	0.93	0.83, 1.05	
Same	51	1.29	0.93, 1.79	126	1	Referent	31	0.74	0.50, 1.10		208	0.90	0.82, 0.99	
Heavier	36	1.16	0.76, 1.77	53	1	Referent	9	0.69	0.34, 1.40		98	0.91	0.80, 1.05	
BMI at enrollment^d^										.43				.16
<25 kg/m^2^	24	1.53	0.96, 2.46	61	1	Referent	21	0.73	0.44, 1.20		106	0.85	0.75, 0.97	
≥25 kg/m^2^	85	1.13	0.88, 1.47	197	1	Referent	57	0.82	0.61, 1.10		339	0.95	0.88, 1.02	

Abbreviation: BMI, body mass index.

^a^ Adjusted for age as the time scale, birth cohort, race and ethnicity, and family income level growing up.

^b^ P for heterogeneity from a joint Wald test of exposure × modifier interaction term(s).

^c^ Eighty‐five participants missing data on weight relative to peers at age 10 years were excluded from this analysis.

^d^ 11 participants missing examiner measurements of BMI at enrollment were excluded from this analysis.

^e^ Not shown. We do not present estimates of association for groups with less than five exposed cases.

Uterine cancer HRs associated with continuous age at thelarche were similar or slightly stronger in magnitude across most sensitivity analyses, ranging from 0.87–0.91 per 1‐year older (Table S1), compared to the original estimate of 0.91. The exception was for the analysis conducted only among the 10,059 participants who reported menarche at age 13 years (the median age in the cohort), where the HR per 1‐year older thelarche was 0.77 (95% CI 0.65–0.91). Relative to thelarche at ages 11–12 years, HRs for thelarche <11 years ranged from 1.14 to 1.79, while HRs ranged from 0.65 to 0.81 for thelarche ≥13 years.

## 4 DISCUSSION

Consistent with prior studies,^4^, ^5^, ^6^, ^26^, ^27^, ^28^, ^29^, ^30^, ^31^, ^32^, ^33^, ^34^ we found that younger age at menarche was associated with increased uterine cancer incidence in a US‐wide prospective cohort. Adding to this literature, we also identified younger age at thelarche as a novel uterine cancer risk factor. These trends were particularly pronounced among women born in 1950 or later, and potentially stronger among pre‐menopausal and younger women. Our findings support the hypothesis that the secular declines in age at pubertal onset^13^, ^14^ may contribute to rising rates of uterine cancer incidence,^7^, ^15^, ^16^ which have been increasing at a faster rate among younger women compared to older age groups.^35^ The steep rate of increase among women <50 years points to a role for early‐life exposures in potentially contributing to this trend.

The associations we observed of earlier thelarche and menarche with increased uterine cancer risk could be explained by earlier and possibly prolonged exposure to unopposed estrogen.^11^ Ages at thelarche and menarche are positively correlated, but girls with earlier thelarche experience a longer time to menarche on average than those with later thelarche, suggesting a longer duration of unopposed estrogen exposure; these patterns are observed in our cohort based on recalled ages^21^ and in prior studies with prospective pubertal timing assessments.^18^ Not surprisingly given their correlation of 0.6, associations of ages at thelarche and menarche with uterine cancer incidence were attenuated when both milestones were included in the same model. It is difficult to disentangle the independent effects of ages at thelarche and menarche, particularly when reliant on recalled data that is likely not reported with the same degree of accuracy and precision as data collected prospectively. Our analysis considering the timing of both milestones jointly suggested that experiencing either milestone at an earlier age was associated with increased risk, while experiencing either at a later age was associated with decreased risk. We did observe a strong inverse association of age at thelarche among the subgroup of women who reported menarche at 13 years of age, which suggests a potential independent effect of earlier thelarche that may reflect a longer duration of early pubertal unopposed estrogen exposure. However, we did not model the time from thelarche to menarche directly due to concerns that measurement error in the recalled ages of each milestone could be compounded in the derivation of the tempo variable, as discussed in depth in our prior analysis of pubertal timing and breast cancer incidence in the cohort.^21^

There is wide variability in the amount of time it takes to achieve regular, ovulatory menstrual cycles.^10^ Recent studies suggest that in the first few years after menarche, there is a gradual transition from anovulatory cycles (no progesterone exposure) to ovulatory cycles with a short (<10 days) luteal phase length (lower progesterone exposure) to ovulatory cycles with a normal (≥10 days) luteal phase length (higher progesterone exposure).^10^, ^36^, ^37^ While an earlier clinical study found that girls with earlier menarche experienced earlier onset of ovulatory cycles,^38^ recent epidemiologic studies using menstrual app data have observed greater time to regularity^39^ and altered menstrual cycle characteristics (e.g., differences in odds of dysmenorrhea, short or long cycle length, and/or cycle variability)^40^ among girls with either early or late menarche (vs. average ages). We did not have data on age at onset of regular cycles, which would have allowed us to more directly explore the hypothesis that a longer duration of unopposed estrogen exposure during puberty is associated with increased uterine cancer risk. However, the onset of regular menstrual cycles is difficult to accurately recall,^41^ and cycle length may be a relatively poor marker of ovulation among adolescents.^37^ Studies with prospective data on ages at thelarche and menarche, detailed menstrual cycle information, and repeated hormone biomarker data across the pubertal transition may be necessary to fully explore how the timing of these markers reflect changes in the hormonal milieu and collectively influence risk of uterine and other hormone‐sensitive cancers.

Endometrial cancers, which make up 90% of uterine cancers,^3^ are typically classified into two types based on differences in histology, clinical outcomes, and potential etiology.^42^, ^43^ Type I tumors include predominantly endometrioid histologic subtypes and are thought to be hormone‐dependent. These typically have better outcomes than type II tumors, which include nonendometrioid subtypes and were previously thought to be hormone‐independent.^44^ However, this dual etiology model has been challenged by studies supporting that hormonal factors (including earlier age at menarche) and metabolic factors are associated in a similar manner with type I and type II tumors.^34^, ^45^, ^46^ We were underpowered to detect associations with incidence of nonendometrioid tumors due to small case counts. While CIs were wider around HRs for nonendometrioid tumors compared to those for endometrioid tumors, the point estimates for ages at thelarche and menarche were inverse and of a similar magnitude for both endometrioid and nonendometrioid endometrial tumors, suggesting that the pubertal hormonal milieu may play a role in the development of both types.

We observed inverse associations of thelarche and menarche with incident uterine cancer only among women born in 1950 or later. This heterogeneity by birth cohort is intriguing and could have several explanations. Causes of the secular decline in pubertal timing remain unclear but are thought to include increases in childhood obesity as well as changing exposures to endocrine‐disrupting chemicals in early life,^47^ which may also influence the hormonal milieu, uterine development, and susceptibility to carcinogenesis.^48^ Alternatively, it is possible that women from earlier birth cohorts may have recalled their timing of pubertal milestones less accurately than women from more recent birth cohorts, which could have resulted in a greater degree of bias toward the null in that stratum. Incident cases among women born before 1950 were more likely to be diagnosed at later ages and after menopause. If early‐life exposures are more strongly linked to younger‐onset uterine cancers, this could also potentially explain the heterogeneity that we observed by birth cohort as women from earlier birth cohorts that were diagnosed at younger ages were more likely to have been diagnosed prior to enrollment and thus excluded from the analytic sample. In analyses stratified by age and time‐varying menopausal status, associations were slightly stronger in magnitude for cancers diagnosed at ≤60 years of age or prior to menopause. While some prior studies observed stronger associations of age at menarche with pre‐menopausal endometrial cancers,^28^, ^29^, ^30^ analyses in the large European Prospective Investigation into Cancer and Nutrition (EPIC) cohort^6^ and in two recent pooled analyses conducted within large consortia did not observe differences by menopausal status.^31^, ^34^ Similarly, these studies did not observe evidence of heterogeneity in the menarche‐endometrial cancer association by age.^6^, ^34^ Earlier menarche as well as thelarche thus appear to influence uterine cancer risk to some degree across the spectrum of age and menopausal status, while additional research is warranted to further examine potential differences in these associations by birth cohort.

Rates of uterine cancer incidence have been increasing at a faster rate among Black compared to White women in the United States, including among younger women.^15^, ^35^, ^49^ We hypothesized that differences in pubertal timing could contribute to these differences in the rate of increase in uterine cancer incidence by race, as the distribution of ages at thelarche and menarche are shifted earlier in US Black girls compared to White girls.^17^, ^18^, ^19^ We observed similar patterns of association with incident uterine cancer among Black and White women in our cohort, though the estimates for Black women were much less precise due to small case counts. While associations did not vary by race, the attributable risk of uterine cancer due to earlier puberty may be higher among Black women due to the higher prevalence of early puberty.

Girls with a higher BMI are more likely to experience earlier thelarche^17^ and menarche^18^ and to have a higher BMI in adulthood.^23^ Associations were very similar for timing of thelarche and menarche in analyses adjusted for weight relative to peers at age 10 years as a potential confounder. However, residual confounding is possible due to our reliance on relative categories of childhood weight reported retrospectively, which prior studies suggest are moderately correlated with actual measures of weight taken in childhood.^50^ Associations were attenuated, particularly for the categories of early thelarche and menarche, in analyses also adjusted for BMI at enrollment, which could potentially reflect additional control for confounding by early‐life body size or indicate partial mediation by adiposity in adulthood. When we stratified by relative childhood weight (lighter, same weight, and heavier than peers) and BMI at enrollment (<25 and ≥25 kg/m^2^), we observed similar, inverse uterine cancer associations for thelarche and menarche across the strata, which suggests that pathways other than adiposity likely play a role in the observed associations.

To our knowledge, this is the first study to examine age at thelarche in relation to uterine cancer risk. Long‐term recall of age at menarche has been found to have good accuracy,^41^ but the validity of recalled age at thelarche is not known. Average age at menarche in our analytic sample (12.7 years) is similar to the average age reported in prior studies with prospective data from women in similar birth cohorts (reviewed in Ref. [18]) but average age at thelarche (12.2 years) is about 1 year later, suggesting that women may have overestimated it.^21^ We categorized the timing of thelarche and menarche as early, average, and late to minimize the potential error in recall of exact ages, but exposure misclassification that was non‐differential by uterine cancer status likely remained and biased the effect estimates toward the null, particularly for age at thelarche. A recent study comparing retrospective reports of pubertal timing relative to peers assessed in adulthood with prospective self‐reports of physical development obtained in adolescence found moderate to high convergence between the retrospective and contemporaneous measures.^51^ Convergence was lowest in girls with early puberty, who were more likely to recall on‐time development as adults.^51^ Our sensitivity analyses that corrected implausible ages at thelarche to address potential recall biases consistently showed a stronger association for early thelarche and uterine cancer than we observed in our primary analyses. Considering secular trends in age at thelarche, our findings warrant replication in contemporary cohorts with prospectively attained puberty and anthropometric data.

While we consider our effect modification analyses to be a strength of the study, we had small case numbers in some strata, limiting the precision of effect estimates and our ability to detect statistical heterogeneity. The Sister Study is a volunteer cohort of participants with a family history of breast cancer. Although breast cancer family history has not been consistently linked with uterine cancer risk,^52^ Sister Study participants may differ from the general population in terms of risk factor profiles—particularly for factors that are associated with risk of both breast and uterine cancers—which may limit the generalizability of our findings.

## 5 CONCLUSIONS

Our findings suggest that younger ages at thelarche and menarche, markers of earlier and potentially prolonged exposure to unopposed estrogen during puberty, may increase susceptibility to uterine carcinogenesis. Future studies with prospective data on ages at thelarche, menarche, and the onset of regular ovulatory cycles, as well as the time intervals between these milestones, could help to refine our understanding of the hormonal mechanisms underlying the observed increased risk and provide additional insights into how secular trends in pubertal timing and tempo may influence rates of uterine and other cancers.

## AUTHOR CONTRIBUTIONS

**Ariayana N. Harrell:** Formal analysis; writing – original draft. **Katie M. O'Brien:** Data curation; writing – review and editing. **Natalie D. Shaw:** Writing – review and editing. **Dale P. Sandler:** Resources; funding acquisition; supervision; writing – review and editing. **Mandy Goldberg:** Conceptualization; methodology; formal analysis; visualization; writing – review and editing.

## FUNDING INFORMATION

This work was supported by the Intramural Research Program of the National Institutes of Health, National Institute of Environmental Health Sciences (Z01‐ES044005 to Dale P. Sandler and ZIAES103399 to Mandy Goldberg). Mandy Goldberg was supported in part by a grant (R00HD110645) from the *Eunice Kennedy Shriver* National Institute of Child Health and Human Development. The contributions of the NIH authors were made as part of their official duties as National Institutes of Health (NIH) federal employees, are in compliance with agency policy requirements, and are considered Works of the United States Government. However, the findings and conclusions presented in this paper are those of the authors and do not necessarily reflect the views of the NIH or the U.S. Department of Health and Human Services.

## CONFLICT OF INTEREST STATEMENT

The authors have no conflicts of interest to disclose.

## ETHICS STATEMENT

All participants provided written informed consent. The Sister Study was approved by the institutional review board of the National Institutes of Health.

## Supporting information


**Data S1.** Supporting Information.

## Data Availability

The data that support the findings of this study are available upon reasonable request as described on the Sister Study website (https://sisterstudy.niehs.nih.gov/English/data-requests.htm). Further information is available from the corresponding author upon request.

## References

[1] National Cancer Institute . Cancer stat facts: uterine cancer. https://seer.cancer.gov/statfacts/html/corp.html. Accessed on 26 March 2025.

[2] Bray F , Laversanne M , Sung H , et al. Global cancer statistics 2022: GLOBOCAN estimates of incidence and mortality worldwide for 36 cancers in 185 countries. CA Cancer J Clin. 2024;74(3):229‐263. 38572751 10.3322/caac.21834

[3] Felix AS , Brinton LA . Cancer progress and priorities: uterine cancer. Cancer Epidemiol Biomarkers Prev. 2018;27(9):985‐994. 30181320 10.1158/1055-9965.EPI-18-0264 PMC6504985

[4] Gong TT , Wang YL , Ma XX . Age at menarche and endometrial cancer risk: a dose‐response meta‐analysis of prospective studies. Sci Rep. 2015;5:14051. 26360785 10.1038/srep14051 PMC4566123

[5] McPherson CP , Sellers TA , Potter JD , Bostick RM , Folsom AR . Reproductive factors and risk of endometrial cancer. The Iowa Women's health study. Am J Epidemiol. 1996;143(12):1195‐1202. 8651218 10.1093/oxfordjournals.aje.a008707

[6] Dossus L , Allen N , Kaaks R , et al. Reproductive risk factors and endometrial cancer: the European Prospective Investigation into Cancer and Nutrition. Int J Cancer. 2010;127(2):442‐451. 19924816 10.1002/ijc.25050

[7] Qiu S , Jiang S , Ye Q , Yang Y , Li X . Global trends and geographical disparities in the incidence of uterine cancer from 1990 to 2021. Eur J Obstet Gynecol Reprod Biol. 2025;311:114066. 40460669 10.1016/j.ejogrb.2025.114066

[8] Abreu AP , Kaiser UB . Pubertal development and regulation. Lancet Diabetes Endocrinol. 2016;4(3):254‐264. 26852256 10.1016/S2213-8587(15)00418-0 PMC5192018

[9] Lacroix AE , Gondal H , Shumway KR , Langaker MD . Physiology, Menarche. StatPearls Publishing LLC; 2025. 29261991

[10] Carlson LJ , Shaw ND . Development of ovulatory menstrual cycles in adolescent girls. J Pediatr Adolesc Gynecol. 2019;32(3):249‐253. 30772499 10.1016/j.jpag.2019.02.119 PMC6570576

[11] Key TJ , Pike MC . The dose‐effect relationship between ‘unopposed’ oestrogens and endometrial mitotic rate: its central role in explaining and predicting endometrial cancer risk. Br J Cancer. 1988;57(2):205‐212. 3358913 10.1038/bjc.1988.44 PMC2246441

[12] Persson I . Estrogens in the causation of breast, endometrial and ovarian cancers – evidence and hypotheses from epidemiological findings. J Steroid Biochem Mol Biol. 2000;74(5):357‐364. 11162945 10.1016/s0960-0760(00)00113-8

[13] Lee Y , Styne D . Influences on the onset and tempo of puberty in human beings and implications for adolescent psychological development. Horm Behav. 2013;64(2):250‐261. 23998669 10.1016/j.yhbeh.2013.03.014

[14] Eckert‐Lind C , Busch AS , Petersen JH , et al. Worldwide secular trends in age at pubertal onset assessed by breast development among girls: a systematic review and meta‐analysis. JAMA Pediatr. 2020;174(4):e195881. 32040143 10.1001/jamapediatrics.2019.5881 PMC7042934

[15] Henley SJ , Miller JW , Dowling NF , Benard VB , Richardson LC . Uterine cancer incidence and mortality – United States, 1999‐2016. MMWR Morb Mortal Wkly Rep. 2018;67(48):1333‐1338. 30521505 10.15585/mmwr.mm6748a1 PMC6329484

[16] Clarke MA , Devesa SS , Harvey SV , Wentzensen N . Hysterectomy‐corrected uterine corpus cancer incidence trends and differences in relative survival reveal racial disparities and rising rates of nonendometrioid cancers. J Clin Oncol. 2019;37(22):1895‐1908. 31116674 10.1200/JCO.19.00151 PMC6675596

[17] Biro FM , Greenspan LC , Galvez MP , et al. Onset of breast development in a longitudinal cohort. Pediatrics. 2013;132(6):1019‐1027. 24190685 10.1542/peds.2012-3773 PMC3838525

[18] Biro FM , Pajak A , Wolff MS , et al. Age of menarche in a longitudinal US cohort. J Pediatr Adolesc Gynecol. 2018;31(4):339‐345. 29758276 10.1016/j.jpag.2018.05.002 PMC6121217

[19] Herman‐Giddens ME , Slora EJ , Wasserman RC , et al. Secondary sexual characteristics and menses in young girls seen in office practice: a study from the pediatric research in office settings network. Pediatrics. 1997;99(4):505‐512. 9093289 10.1542/peds.99.4.505

[20] Sandler DP , Hodgson ME , Deming‐Halverson SL , et al. The Sister Study cohort: baseline methods and participant characteristics. Environ Health Perspect. 2017;125(12):127003. 29373861 10.1289/EHP1923 PMC5963586

[21] Goldberg M , D'Aloisio AA , O'Brien KM , Zhao S , Sandler DP . Pubertal timing and breast cancer risk in the Sister Study cohort. Breast Cancer Res. 2020;22(1):112. 33109223 10.1186/s13058-020-01326-2 PMC7590599

[22] Harvey SV , Wentzensen N , Bertrand K , et al. Associations of life course obesity with endometrial cancer in the epidemiology of endometrial cancer consortium (E2C2). Int J Epidemiol. 2023;52(4):1086‐1099. 37029916 10.1093/ije/dyad046 PMC10396409

[23] Stovitz SD , Pereira MA , Vazquez G , Lytle LA , Himes JH . The interaction of childhood height and childhood BMI in the prediction of young adult BMI. Obesity (Silver Spring). 2008;16(10):2336‐2341. 18719630 10.1038/oby.2008.359 PMC2747360

[24] Goldberg M , D'Aloisio AA , O'Brien KM , Zhao S , Sandler DP . Early‐life exposures and age at thelarche in the Sister Study cohort. Breast Cancer Res. 2021;23(1):111. 34895281 10.1186/s13058-021-01490-z PMC8666031

[25] Lin DY , Wei L‐J . The robust inference for the Cox proportional hazards model. J Am Stat Assoc. 1989;84(408):1074‐1078.

[26] Wernli KJ , Ray RM , Gao DL , De Roos AJ , Checkoway H , Thomas DB . Menstrual and reproductive factors in relation to risk of endometrial cancer in Chinese women. Cancer Causes Control. 2006;17(7):949‐955. 16841262 10.1007/s10552-006-0034-6

[27] Kvåle G , Heuch I , Ursin G . Reproductive factors and risk of cancer of the uterine corpus: a prospective study. Cancer Res. 1988;48(21):6217‐6221. 3167867

[28] Sponholtz TR , Palmer JR , Rosenberg L , Hatch EE , Adams‐Campbell LL , Wise LA . Reproductive factors and incidence of endometrial cancer in U.S. Black women. Cancer Causes Control. 2017;28(6):579‐588. 28361447 10.1007/s10552-017-0880-4 PMC5634741

[29] La Vecchia C , Franceschi S , Decarli A , Gallus G , Tognoni G . Risk factors for endometrial cancer at different ages. J Natl Cancer Inst. 1984;73(3):667‐671. 6590913

[30] Xu WH , Xiang YB , Ruan ZX , et al. Menstrual and reproductive factors and endometrial cancer risk: results from a population‐based case‐control study in urban Shanghai. Int J Cancer. 2004;108(4):613‐619. 14696129 10.1002/ijc.11598

[31] Katagiri R , Iwasaki M , Abe SK , et al. Reproductive factors and endometrial cancer risk among women. JAMA Netw Open. 2023;6(9):e2332296. 37669051 10.1001/jamanetworkopen.2023.32296 PMC10481237

[32] Ghanbari Andarieh M , Agajani Delavar M , Moslemi D , Esmaeilzadeh S . Risk factors for endometrial cancer: results from a hospital‐based case‐control study. Asian Pac J Cancer Prev. 2016;17(10):4791‐4796. 27910901 10.22034/APJCP.2016.17.10.4791 PMC5454633

[33] Cote ML , Alhajj T , Ruterbusch JJ , et al. Risk factors for endometrial cancer in Black and White women: a pooled analysis from the epidemiology of endometrial cancer consortium (E2C2). Cancer Causes Control. 2015;26(2):287‐296. 25534916 10.1007/s10552-014-0510-3 PMC4528374

[34] Peeri NC , Bertrand KA , Na R , et al. Understanding risk factors for endometrial cancer in young women. J Natl Cancer Inst. 2025;117(1):76‐88. 39235934 10.1093/jnci/djae210 PMC11717423

[35] Liu L , Habeshian TS , Zhang J , et al. Differential trends in rising endometrial cancer incidence by age, race, and ethnicity. JNCI Cancer Spectr. 2023;7(1):pkad001. 36625534 10.1093/jncics/pkad001 PMC9904185

[36] Zhang K , Pollack S , Ghods A , et al. Onset of ovulation after menarche in girls: a longitudinal study. J Clin Endocrinol Metab. 2008;93(4):1186‐1194. 18252789 10.1210/jc.2007-1846 PMC2291492

[37] Sun BZ , Kangarloo T , Adams JM , et al. Healthy post‐menarchal adolescent girls demonstrate multi‐level reproductive axis immaturity. J Clin Endocrinol Metab. 2019;104(2):613‐623. 30289507 10.1210/jc.2018-00595 PMC6325170

[38] Apter D , Vihko R . Early menarche, a risk factor for breast cancer, indicates early onset of ovulatory cycles. J Clin Endocrinol Metab. 1983;57(1):82‐86. 6222061 10.1210/jcem-57-1-82

[39] Wang Z , Asokan G , Onnela JP , et al. Menarche and time to cycle regularity among individuals born between 1950 and 2005 in the US. JAMA Netw Open. 2024;7(5):e2412854. 38809557 10.1001/jamanetworkopen.2024.12854 PMC11137638

[40] Harley KG , Watson A , Robertson S , Vitzthum VJ , Shea A . Menstrual cycle characteristics of U. S. adolescents according to gynecologic age and age at menarche. J Pediatr Adolesc Gynecol. 2024;37(4):419‐425. 38570085 10.1016/j.jpag.2024.03.005

[41] Must A , Phillips SM , Naumova EN , et al. Recall of early menstrual history and menarcheal body size: after 30 years, how well do women remember? Am J Epidemiol. 2002;155(7):672‐679. 11914195 10.1093/aje/155.7.672

[42] Bokhman JV . Two pathogenetic types of endometrial carcinoma. Gynecol Oncol. 1983;15(1):10‐17. 6822361 10.1016/0090-8258(83)90111-7

[43] Sherman ME . Theories of endometrial carcinogenesis: a multidisciplinary approach. Mod Pathol. 2000;13(3):295‐308. 10757340 10.1038/modpathol.3880051

[44] Suarez AA , Felix AS , Cohn DE . Bokhman redux: endometrial cancer “types” in the 21st century. Gynecol Oncol. 2017;144(2):243‐249. 27993480 10.1016/j.ygyno.2016.12.010

[45] Setiawan VW , Yang HP , Pike MC , et al. Type I and II endometrial cancers: have they different risk factors? J Clin Oncol. 2013;31(20):2607‐2618. 23733771 10.1200/JCO.2012.48.2596 PMC3699726

[46] Yang HP , Wentzensen N , Trabert B , et al. Endometrial cancer risk factors by 2 main histologic subtypes: the NIH‐AARP diet and health study. Am J Epidemiol. 2013;177(2):142‐151. 23171881 10.1093/aje/kws200 PMC3590033

[47] Parent AS , Teilmann G , Juul A , Skakkebaek NE , Toppari J , Bourguignon JP . The timing of normal puberty and the age limits of sexual precocity: variations around the world, secular trends, and changes after migration. Endocr Rev. 2003;24(5):668‐693. 14570750 10.1210/er.2002-0019

[48] Mallozzi M , Leone C , Manurita F , Bellati F , Caserta D . Endocrine disrupting chemicals and endometrial cancer: an overview of recent laboratory evidence and epidemiological studies. Int J Environ Res Public Health. 2017;14(3):334. 28327540 10.3390/ijerph14030334 PMC5369169

[49] Wijayabahu AT , Shiels MS , Arend RC , Clarke MA . Uterine cancer incidence trends and 5‐year relative survival by race/ethnicity and histology among women under 50 years. Am J Obstet Gynecol. 2024;231(5):526.e1‐526.e22. 10.1016/j.ajog.2024.06.026 PMC11499002 38925206

[50] Casey VA , Dwyer JT , Coleman KA , Krall EA , Gardner J , Valadian I . Accuracy of recall by middle‐aged participants in a longitudinal study of their body size and indices of maturation earlier in life. Ann Hum Biol. 1991;18(2):155‐166. 2024949 10.1080/03014469100001492

[51] Chaku N , Berenbaum SA , Qian Y , et al. Pubertal timing in adolescence and adulthood: relations among contemporaneous and retrospective measures. Dev Psychol. 2025;61(5):928‐943. 39250300 10.1037/dev0001784 PMC11890207

[52] Win AK , Reece JC , Ryan S . Family history and risk of endometrial cancer: a systematic review and meta‐analysis. Obstet Gynecol. 2015;125(1):89‐98. 25560109 10.1097/AOG.0000000000000563

