# Supplementary material for: Pubertal timing and incident uterine cancer in the Sister Study cohort
Source: Int J Cancer. 2025 Dec 24;158(11):2841–52. doi: 10.1002/ijc.70310 (PMC13047254; doi:10.1002/ijc.70310)

Pubertal timing and incident uterine cancer in the Sister Study cohort

Ariayana N. Harrell, Katie M. O'Brien, Natalie D. Shaw, Dale P. Sandler, Mandy Goldberg

Table of Contents

Table S1. Sensitivity analyses for the association of timing of thelarche and incident uterine cancer in the Sister Study……………………………………………………………………..………2

Figure S1. Flow chart of eligible study population……………………………………...………..4

Table S1. Sensitivity analyses for the association of timing of thelarche and incident uterine cancer in the Sister Study

			Age at thelarche[a]											
			<11 years			11-12 years			≥13 years			Continuous (per 1-year older)		
Analysis	n in model	Person-years	n cases	HR	95% CI	n cases	HR	95% CI	n cases	HR	95% CI	n cases	HR	95% CI
Full cohort	34,152	422,439	68	1.15	0.88, 1.50	235	1	Referent	142	0.75	0.61, 0.92	445	0.91	0.85, 0.97
Imputing implausible ages at thelarche:														
Re-assigning age at thelarche to be 1 year earlier than reported for participants who originally reported thelarche <1 year before menarche	34,152	422,439	108	1.32	1.05, 1.65	253	1	Referent	84	0.81	0.64, 1.04	445	0.90	0.84, 0.96
Re-assigning age at thelarche to be 2 years earlier than reported for participants who originally reported thelarche <1 year before menarche	34,152	422,439	184	1.26	1.03, 1.53	212	1	Referent	49	0.72	0.53, 0.99	445	0.91	0.85, 0.97
Using MI[b] to impute age at thelarche for participants who reported thelarche <1 year before menarche	34,152	422,439	156[c]	1.26	0.99, 1.59	239[c]	1	Referent	50[c]	0.78	0.55, 1.10	445	0.90	0.83, 0.97
Restricting study population to informative subgroups:														
Participants who reported thelarche at least 1 year before menarche	15,846	195,955	52	1.27	0.91, 1.76	119	1	Referent	31	0.68	0.46, 1.01	202	0.87	0.79, 0.97
Participants who reported thelarche at ages 8-14 years	31,632	391,844	67	1.14	0.87, 1.49	235	1	Referent	121	0.78	0.62, 0.96	423	0.91	0.85, 0.98

Participants who reported menarche at age 13 years	10,059	125,494	10	1.79	0.93, 3.45	73	1	Referent	49	0.65	0.45, 0.93	132	0.77	0.65, 0.91

MI: Multiple imputation

[a]Adjusted for age as the time scale and for birth cohort, race and ethnicity, and family income level growing up as covariates

[b]We set age at thelarche to missing for participants who reported that thelarche occurred <1 year before menarche and used multiple imputation (MI) to impute age at thelarche for these participants under the assumption that age at thelarche was missing at random, conditional on the covariates in the MI model. The imputation model included the crude cumulative hazard estimate at the age at diagnosis or censor, uterine cancer case status, age at baseline, age at menarche, birth cohort, race and ethnicity, family income level growing up, relative weight at age 10 years, BMI at enrollment, and early-life exposures that we previously found to be associated with age at thelarche (maternal age at participant’s birth, multiple birth, firstborn, maternal smoking during pregnancy, maternal pre-pregnancy or gestational diabetes, maternal gestational hypertensive disorder during pregnancy, and gestational age at birth, see Goldberg et al, *Breast Cancer Res* 2021 for more details). We used chained equations with the predictive mean matching method to impute age at thelarche as a continuous variable, generating 20 imputed datasets. We categorized age at thelarche using the imputed continuous variable. We ran the multivariable-adjusted Cox proportional hazards model in each imputed dataset and combined the parameter estimates using Rubin’s rules.

[c]The mean number of cases per category of thelarche across the 20 imputed datasets is presented. The number of cases in each thelarche category ranged from 145-164 for <11 years, 227-249 for 11-12 years, and 45-58 for >13 years across the 20 imputed datasets.

Figure S1. Flow chart of eligible study population.

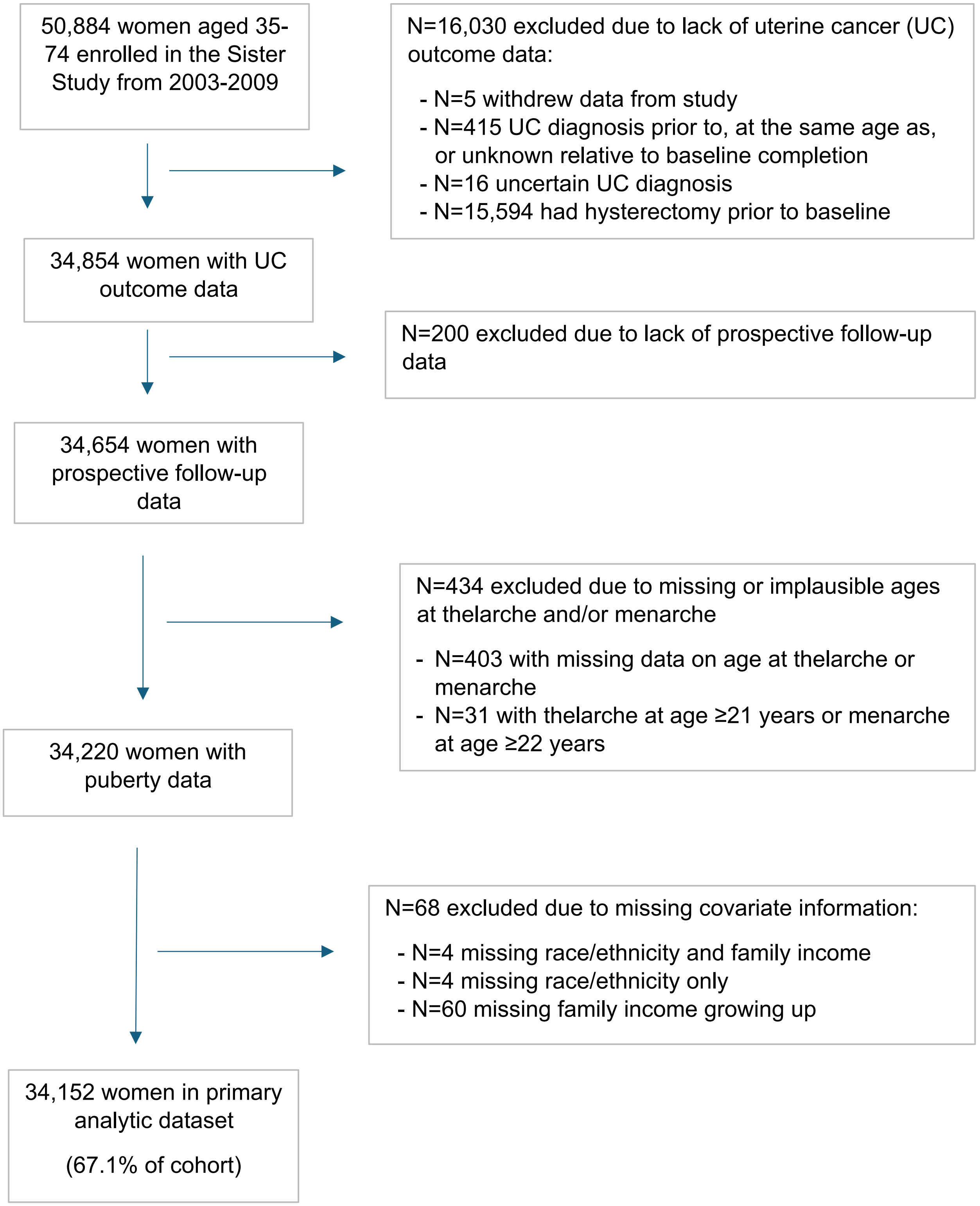

Supplement: Supplementary file 1 — Data S1. Supporting Information. [file IJC-158-2841-s001.pdf]
